# Supplementary material for: Interleukin-8 in Colorectal Cancer: A Systematic Review and Meta-Analysis of Its Potential Role as a Prognostic Biomarker
Source: Biomedicines. 2022 Oct 19;10(10):2631. doi: 10.3390/biomedicines10102631 (PMC9599846; doi:10.3390/biomedicines10102631)
Supplement: Supplementary file 1 [file biomedicines-10-02631-s001.zip › Supplementary_Material_Proof.pdf]

Supplementary Material

1 Supplementary Tables

Supplementary Table S1. Bio-pathological characteristics of CRC patients.

Patient Characteristics	Number of Patients	%
Age at diagnosis (median, range)	67 (35-91)	
Gender		
Male	104	62
Female	64	38
Tumor location		
Right colon	52	31
Left colon	21	13
Rectum	50	30
Sigma+Cecum	35	21
Transverse colon	10	6
TNM		
T3-4	148	88
N1-2	86	51

M1	36	21
Stage		
I	13	8
II	57	34
III	62	37
IV	36	21
Grading		
G1	2	1
G2	135	80
G3	31	18
PTEN status		
Gene wt/ protein +	108	64
Gene wt/ protein -	53	32
Gene mut/ protein +	5	3
Gene mut/ protein -	2	1
RAS/RAF mutational status		
KRAS-mut	75	45
NRAS-mut	10	6
HRAS-mut	1	1

BRAF-mut	11	7
RAS/RAF wt	71	42
Other mutations (>5% frequency)		
APC	83	49
TP53	69	41
KDR	55	33
PIK3CA	55	33
c-KIT	29	17
SMAD4	15	9
ATM	13	8
FBXW7	12	7
PDGFRA	11	7
SMARCB1	10	6
JAK3	9	5
Total n° of mutations (median, range)	3 (0-7)	
Surgery*		
T	168	100
M	37	22
Systemic treatment**		

Neoadjuvant	14	8
Adjuvant	77	46
Metastatic		
I line	60	36
II line or more	39	23
Median FU in months (range)	50 (1-167)	

*Percentages may add up to >100 because the same patient could have undergone surgery on both T/N and M. ** Percentages may add up to >100 because the same patient could have undergone systemic treatment in more than one setting

Supplementary Table S2. Characteristics of included studies.

First Author	Year	Cohort size*	Reference trial	Definition of the study cohort	IL-8 source	IL-8 cutoff	Main findings	Ref
Kopetz	2011	40 (93)	-	I-line mCRC pts, w RECIST measurable disease, all treated with FOLFIRI+B in a prospective phase II setting	Plasma	Median (3.7 pg/mL)	High IL-8 associated with shorter PFS (11 *vs* 15.1 months; *p*=0.03)	[1]
Liu	2013	38 (76)	NCT00416494	I-line mCRC pts, w RECIST measurable disease, all treated with CapeOx+B in a	Platelet-poor plasma	Median (53 pg/mL)	High IL-8 associated with worse OS at univariate analysis (HR	[2]

				prospective phase II setting			2.2; 95% CI 1.06-4.4; *p*=0.03)	
Spencer	2013	582 (54)	HORIZON II (NCT00399035)	I-line mCRC pts, receiving FOLFOX/XELOX w (*N*=330 pts) or w/o (*N*=252 pts) cediranib within a double-blind phase III study	Serum	Median	High IL-8 significantly associated with worse PFS and OS across treatment arms	[3]
Bruhn	2013	196 (41)	MAX	I-line mCRC pts, randomly assigned to receive capecitabine (*N*=60 pts); capecitabine and bevacizumab (*N*=68 pts) and capecitabine, B and mitomycin C (*N*=68 pts)	Tissue-derived protein lysates	Median<br><br>(1.21 pg/mL)	No significant differences in ORR, PFS, OS by IL-8 levels; no significant interaction between IL-8 levels and B benefit	[4]
Chen	2015	176 (41)	-	mCRC pts who had completed oxaliplatin-based I-line and irinotecan-based II-line CHT;<br><br>peripheral blood samples were collected at baseline, before the start of I-line	Serum	Value closest to the maximum sensitivity and specificity for death by ROC curve	High IL-8 significantly associated with worse OS at multivariate analysis (HR 2.056, *p*=0.003)	[5]
Hamilt on	2014	69	-	mCRC pts with liver-only metastases (synchronous 52%, metachronous 48%) referred for surgery; most	Serum	n.s.	High IL-8 associated with worse OS (HR 4.96, 95% CI 1.35-17.6,	[6]

				pts neoadjuvant/adjuvant chemotherapy			*p*=0.014) before adjustment	
Tabern ero	2015	199 (26)	CORRECT	mCRC patients receiving regorafenib or placebo within a randomized trial; data on the impact of IL-8 on outcome only available for 199 patients in the placebo group (total placebo patients: 255)	Plasma	Median, best fit, ROC curve	No significant interactions between IL-8 levels and benefit from treatment; IL-8 prognostic for both PFS (HR 1.63, 95% CI 1.22-2.18, p<0.001, using median as cut-off) and OS (HR 3.48, 95% CI 2.39-5.06, p<0.001, using median as cut-off) in the placebo group	[7]
Di Salvato re	2017	50 (21)	-	I-line, *RAS*-mutant, mCRC pts, treated with FOLFOX/B	Serum	Optimal cutoff by ROC curve based on PFS (18.25 pg/mL; median: 17 pg/mL)	High IL-8 significantly associated with worse PFS (HR 2.9, 95% CI 1.53-5.75, *p*=0.0014) and OS (HR 3.2, 95% CI 1.7-5.9, *p*=0.0002) at multivariate analysis	[8]
Marisi	2018	58 (13)	ITACa (NCT01878422)	I-line, mCRC pts, treated with either FOLFOX or FOLFIRI plus B	Serum	Optimal cutoff by ROC curve based on PFS (145 pg/mL	High IL-8 significantly associated with worse PFS (HR 7.39, 95% CI 2.79-19.83, *p*<0.0001) and OS (HR 7.68, 95% CI 2.59-22.77, *p*<0.001)	[9]

Varkaris	2019	211	-	mCRC pts with unresectable (*N*=121 pts) or potentially resectable (*N*=90 pts) metastases at presentation, treated with standard CHT (no details on CHT regimens provided)	Plasma	Median	High IL-8 not prognostic for PFS/OS individually, but as part of an inflammatory signature also comprising IL-6 and miR-21	[10]
Park	2020	400	-	Patients undergoing surgery for primary CRC at the National Cancer Center, Korea (2009-2010)	Serum	Median	High IL-8 independently associated with worse PFS (HR 1.82, 95% CI 1.19–2.76, $p<0.1$) and OS (HR 2.33, 95% CI 1.32–4.11, $p<0.1$)	**[11]**
Suenaga	2020	125	-	mCRC patients undergoing CHT+B first-line (FL cohort, n=71) or salvage regorafenib (SL cohort, n=54)	Serum	Optimal cutoff by ROC curve based on association with liver metastases (15.1 pg/mL)	High IL-8 independently associated with worse OS in the combined cohort (HR 3.24, 95% CI 1.47–7.16, $p=0.004$)	**[12]**

*Numbers between brackets indicate the percentage of patients tested for IL-8 expression among those enrolled in the original/reference clinical series (where applicable).
B, bevacizumab; CHT, chemotherapy; HR, hazard ratio; IL, interleukin; mCRC, metastatic colorectal cancer; ns, not specified; ORR, objective response rate; OS, overall survival; PFS, progression-free survival; pts, patients; ROC, receiver operating characteristic; w, with; w/o, without; 95% CI, 95% confidence intervals.

Supplementary Table S3. Quality assessment by means of Newcastle-Ottawa Scale (NOS) tool for cohort studies.

	Liu Y et al., 2013	Hamilton TD et al., 2014	Kopetz S et al., 2011	Marisi G et al., 2018	Spencer SKM et al., 2013	Tabernero J et al., 2015	Suenaga M et al., 2020	Bruhn MA et al., 2013	Di Salvatore M et al., 2017	Chen ZY et al., 2015	Varkaris A et al., 2019	Park JW et al., 2020
**Selection**												
Representativeness of the exposed cohort	0	0	0	0	1	0	0	1	1	1	1	1
Selection of the non-exposed cohort	0	1	1	0	1	1	1	1	1	0	1	1
Ascertainment of exposure	1	0	1	1	1	1	1	0	0	1	1	1
Demonstration that outcome of interest was not present at start of study	1	1	1	1	1	1	1	1	1	1	1	1
**Comparability**												
Comparability of cohorts on the bases of	0	1	0	0	1	0	0	1	1	1	1	1

the design or analysis												
**Outcome**												
Assessment of outcome	1	0	1	1	0	1	1	1	1	1	1	1
Was follow-up long enough for outcomes to occur	0	0	0	1	0	0	1	1	1	1	1	1
Adequacy of follow-up of cohorts	0	0	0	1	0	1	0	1	1	1	1	1
**Total score per study**	**3**	**3**	**4**	**5**	**5**	**5**	**5**	**7**	**7**	**7**	**8**	**8**

2 Supplementary Figures and Figure Legends

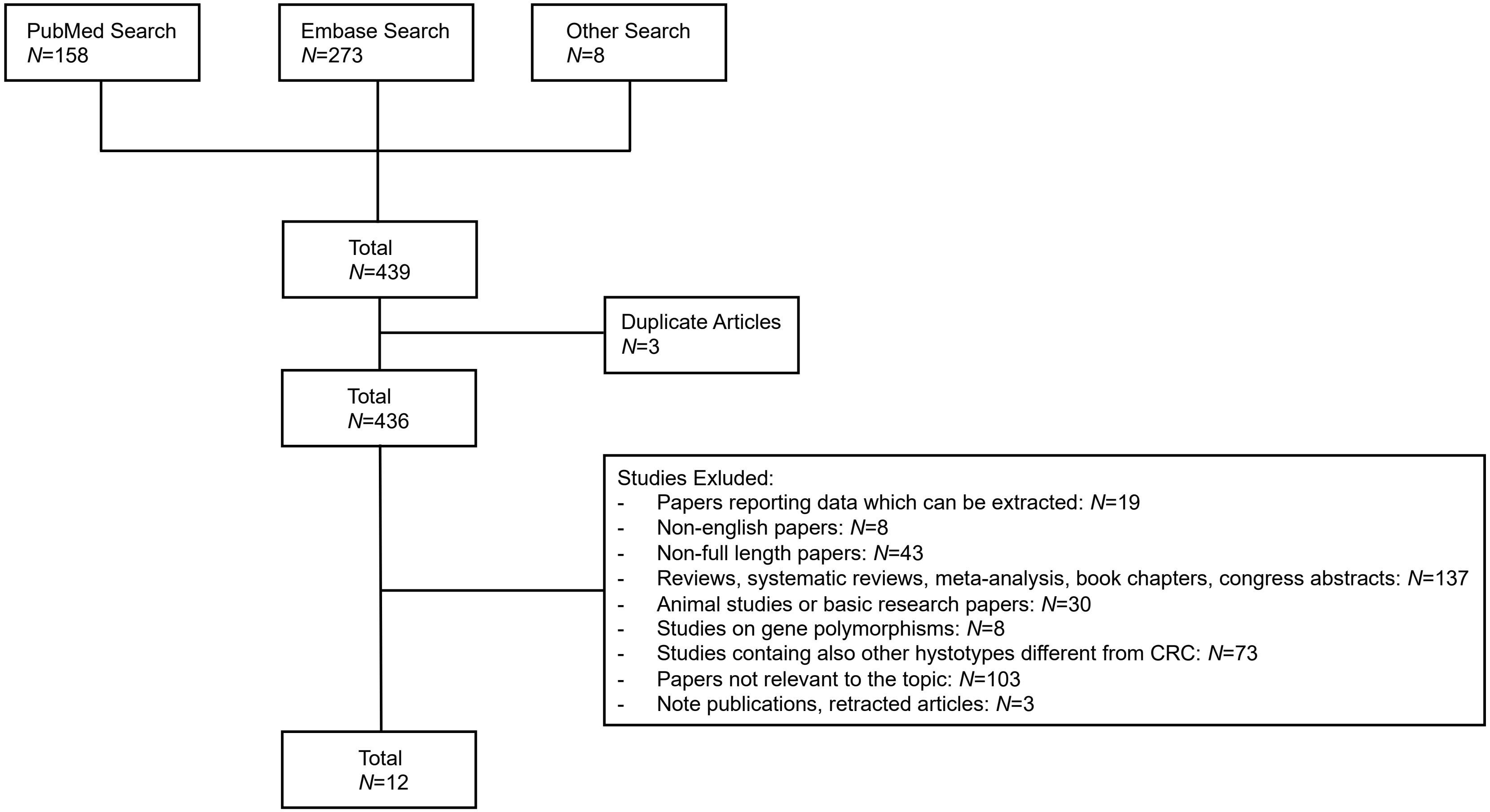

Supplementary Figure S1. Selection of included papers. PRISMA flowchart of the selection of relevant publications included in the meta-analysis. N indicates the number of papers.

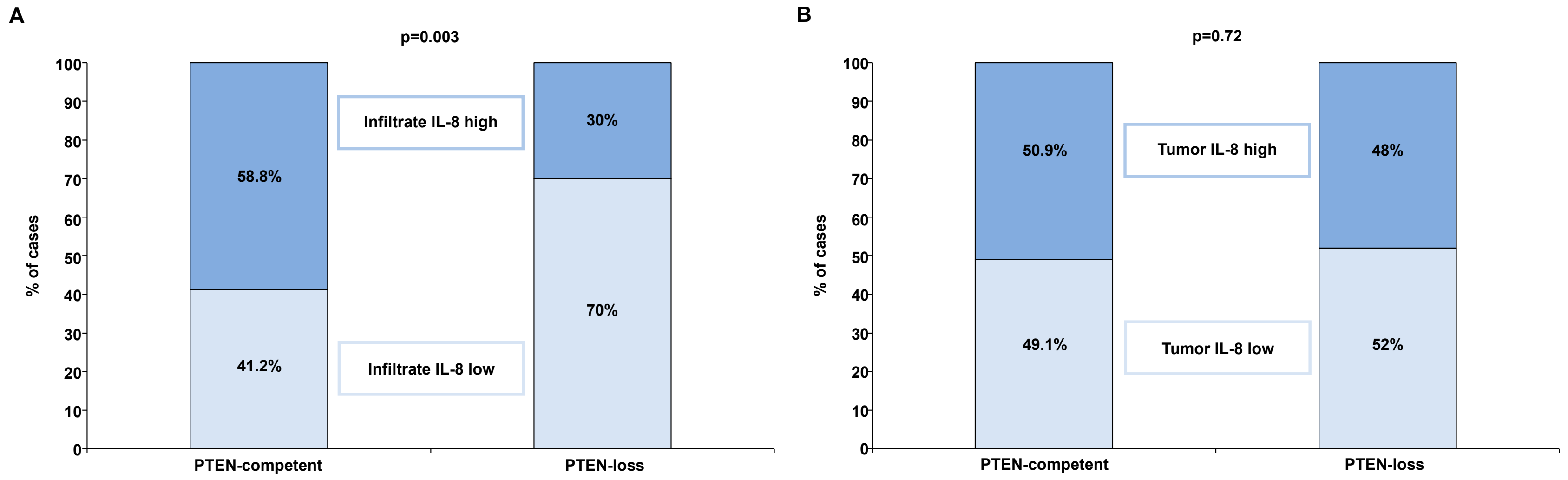

Supplementary Figure S2. Analysis of PTEN status and IL-8 expression. A. Distribution of PTEN competent and PTEN-loss patients according to IL-8 infiltrate levels. **B.** Distribution of PTEN competent and PTEN-loss patients according to IL-8 tumor levels.

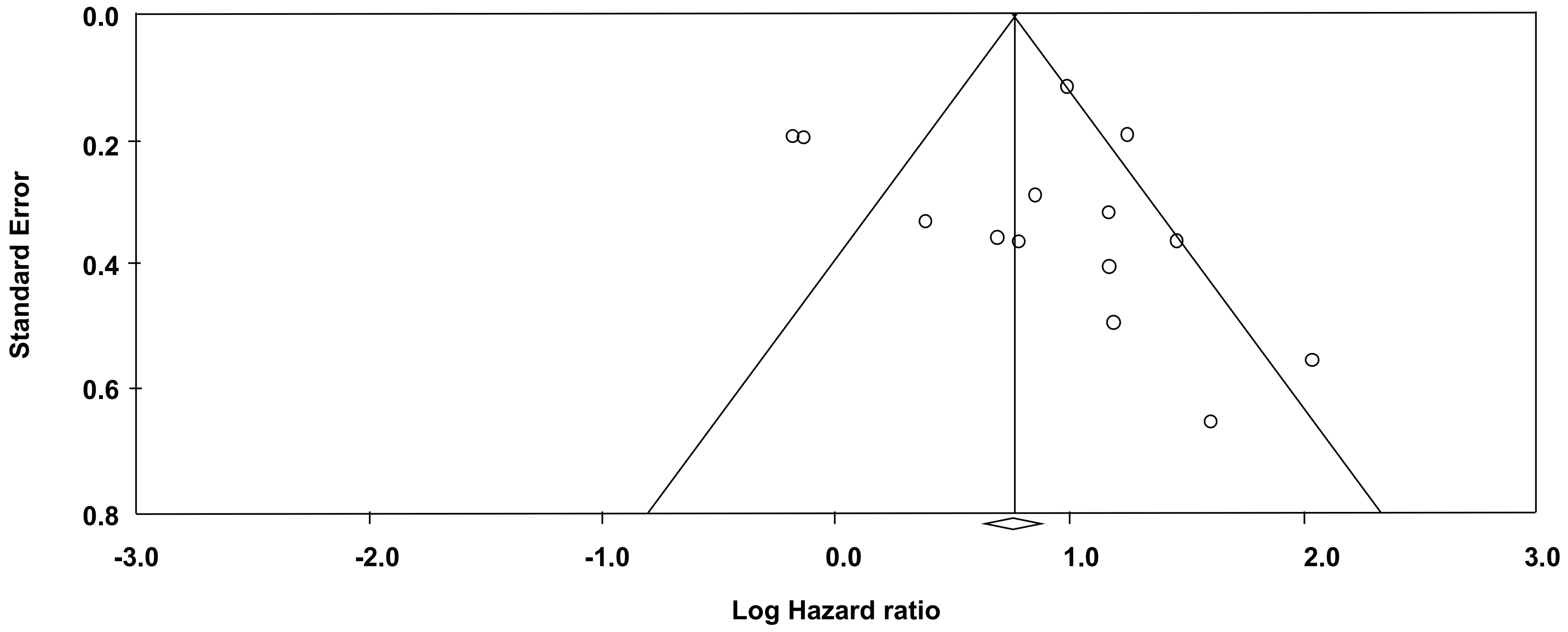

Supplementary Figure S3. Funnel plot illustration of the distribution of studies reporting OS data. The funnel plot was visually asymmetrical towards positive associations, suggesting for the presence of small-study effect.

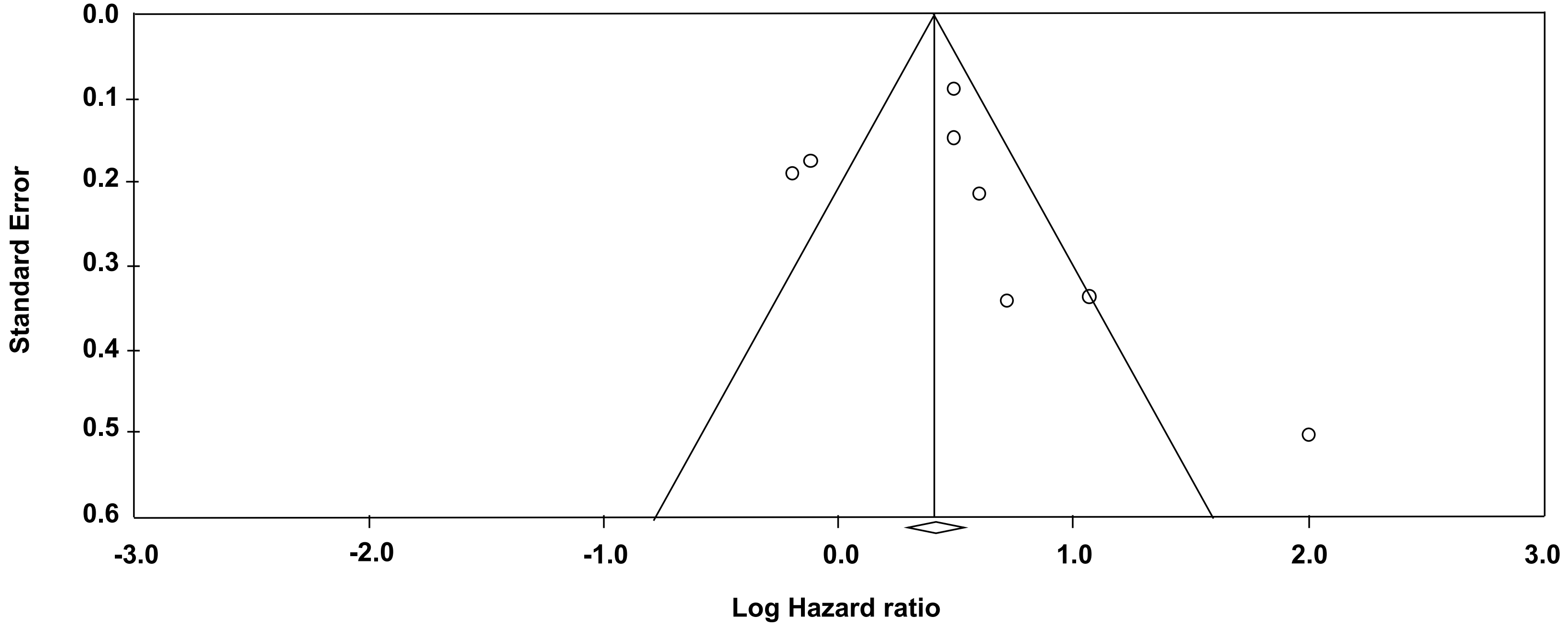

Supplementary Figure S4. Funnel plot illustration of the distribution of studies reporting PFS data. The funnel plot was visually asymmetrical towards positive associations, suggesting for the presence of small-study effect.

3 References

1. Kopetz, S.; Hoff, P.M.; Morris, J.S.; Wolff, R.A.; Eng, C.; Glover, K.Y.; Adinin, R.; Overman, M.J.; Valero, V.; Wen, S., et al. Phase II trial of infusional fluorouracil, irinotecan, and bevacizumab for metastatic colorectal cancer: efficacy and circulating angiogenic biomarkers associated with therapeutic resistance. *J Clin Oncol* **2010**, *28*, 453-459, doi:10.1200/JCO.2009.24.8252.

2. Liu, Y.; Starr, M.D.; Bulusu, A.; Pang, H.; Wong, N.S.; Honeycutt, W.; Amara, A.; Hurwitz, H.I.; Nixon, A.B. Correlation of angiogenic biomarker signatures with clinical outcomes in metastatic colorectal cancer patients receiving capecitabine, oxaliplatin, and bevacizumab. *Cancer Med* **2013**, *2*, 234-242, doi:10.1002/cam4.71.

3. Spencer, S.K.; Pommier, A.J.; Morgan, S.R.; Barry, S.T.; Robertson, J.D.; Hoff, P.M.; Jurgensmeier, J.M. Prognostic/predictive value of 207 serum factors in colorectal cancer treated with cediranib and/or chemotherapy. *Br J Cancer* **2013**, *109*, 2765-2773, doi:10.1038/bjc.2013.649.
4. Bruhn, M.A.; Townsend, A.R.; Khoon Lee, C.; Shivasami, A.; Price, T.J.; Wrin, J.; Arentz, G.; Tebbutt, N.C.; Hocking, C.; Cunningham, D., et al. Proangiogenic tumor proteins as potential predictive or prognostic biomarkers for bevacizumab therapy in metastatic colorectal cancer. *Int J Cancer* **2014**, *135*, 731-741, doi:10.1002/ijc.28698.
5. Chen, Z.Y.; He, W.Z.; Peng, L.X.; Jia, W.H.; Guo, R.P.; Xia, L.P.; Qian, C.N. A prognostic classifier consisting of 17 circulating cytokines is a novel predictor of overall survival for metastatic colorectal cancer patients. *Int J Cancer* **2015**, *136*, 584-592, doi:10.1002/ijc.29017.
6. Hamilton, T.D.; Leugner, D.; Kopciuk, K.; Dixon, E.; Sutherland, F.R.; Bathe, O.F. Identification of prognostic inflammatory factors in colorectal liver metastases. *BMC Cancer* **2014**, *14*, 542, doi:10.1186/1471-2407-14-542.
7. Tabernero, J.; Lenz, H.J.; Siena, S.; Sobrero, A.; Falcone, A.; Ychou, M.; Humblet, Y.; Bouche, O.; Mineur, L.; Barone, C., et al. Analysis of circulating DNA and protein biomarkers to predict the clinical activity of regorafenib and assess prognosis in patients with metastatic colorectal cancer: a retrospective, exploratory analysis of the CORRECT trial. *Lancet Oncol* **2015**, *16*, 937-948, doi:10.1016/S1470-2045(15)00138-2.
8. Di Salvatore, M.; Pietrantonio, F.; Orlandi, A.; Del Re, M.; Berenato, R.; Rossi, E.; Caporale, M.; Guarino, D.; Martinetti, A.; Basso, M., et al. IL-8 and eNOS polymorphisms predict bevacizumab-based first line treatment outcomes in RAS mutant metastatic colorectal cancer patients. *Oncotarget* **2017**, *8*, 16887-16898, doi:10.18632/oncotarget.14810.
9. Marisi, G.; Scarpi, E.; Passardi, A.; Nanni, O.; Pagan, F.; Valgiusti, M.; Casadei Gardini, A.; Neri, L.M.; Frassineti, G.L.; Amadori, D., et al. IL-8 and thrombospondin-1 as prognostic markers in patients with metastatic colorectal cancer receiving bevacizumab. *Cancer Manag Res* **2018**, *10*, 5659-5666, doi:10.2147/CMAR.S181570.
10. Varkaris, A.; Katsiampoura, A.; Davis, J.S.; Shah, N.; Lam, M.; Frias, R.L.; Ivan, C.; Shimizu, M.; Morris, J.; Menter, D., et al. Circulating inflammation signature predicts overall survival and relapse-free survival in metastatic colorectal cancer. *Br J Cancer* **2019**, *120*, 340-345, doi:10.1038/s41416-018-0360-y.
11. Park, J.W.; Chang, H.J.; Yeo, H.Y.; Han, N.; Kim, B.C.; Kong, S.Y.; Kim, J.; Oh, J.H. The relationships between systemic cytokine profiles and inflammatory markers in colorectal cancer and the prognostic significance of these parameters. *Br J Cancer* **2020**, *123*, 610-618, doi:10.1038/s41416-020-0924-5.
12. Suenaga, M.; Mashima, T.; Kawata, N.; Wakatsuki, T.; Dan, S.; Seimiya, H.; Yamaguchi, K. Serum IL-8 level as a candidate prognostic marker of response to anti-angiogenic therapy for metastatic colorectal cancer. *Int J Colorectal Dis* **2021**, *36*, 131-139, doi:10.1007/s00384-020-03748-y.
